# Engineering Built‐In Electric Field Microenvironment of CQDs/g‐C_3_N_4_ Heterojunction for Efficient Photocatalytic CO_2_ Reduction

**DOI:** 10.1002/advs.202403607

**Published:** 2024-05-10

**Authors:** Yun Xu, Weidong Hou, Kai Huang, Huazhang Guo, Zeming Wang, Cheng Lian, Jiye Zhang, Deli Wu, Zhendong Lei, Zheng Liu, Liang Wang

**Affiliations:** ^1^ Institute of Nanochemistry and Nanobiology School of Environmental and Chemical Engineering Shanghai University Shanghai 200444 P. R. China; ^2^ State Key Laboratory of Chemical Engineering Shanghai Engineering Research Center of Hierarchical Nanomaterials and School of Chemistry and Molecular Engineering East China University of Science and Technology Shanghai 200237 P. R. China; ^3^ School of Materials Science and Engineering Shanghai University 99 Shangda Road Shanghai 200444 P. R. China; ^4^ College of Environmental & Engineering Tongji University Shanghai 200092 P. R. China; ^5^ School of Materials Science and Engineering Nanyang Technological University 50 Nanyang Avenue Singapore 639798 Singapore

**Keywords:** built‐in electric field, carbon quantum dots, charge migration, CO_2_ photoreduction, heterojunction

## Abstract

Graphitic carbon nitride (CN), as a nonmetallic photocatalyst, has gained considerable attention for its cost‐effectiveness and environmentally friendly nature in catalyzing solar‐driven CO_2_ conversion into valuable products. However, the photocatalytic efficiency of CO_2_ reduction with CN remains low, accompanied by challenges in achieving desirable product selectivity. To address these limitations, a two‐step hydrothermal‐calcination tandem synthesis strategy is presented, introducing carbon quantum dots (CQDs) into CN and forming ultra‐thin CQD/CN nanosheets. The integration of CQDs induces a distinct work function with CN, creating a robust interface electric field after the combination. This electric field facilitates the accumulation of photoelectrons in the CQDs region, providing an abundant source of reduced electrons for the photocatalytic process. Remarkably, the CQD/CN nanosheets exhibit an average CO yield of 120 µmol g^−1^, showcasing an outstanding CO selectivity of 92.8%. The discovery in the work not only presents an innovative pathway for the development of high‐performance photocatalysts grounded in non‐metallic CN materials employing CQDs but also opens new avenues for versatile application prospects in environmental protection and sustainable cleaning energy.

## Graphical abstract

Embedding nitrogen‐rich CQDs into ultra‐thin g‐C_3_N_4_ nanosheets led to the construction of potent interfacial electric fields, thereby amplifying visible light absorption, and accelerating photoelectron transport. The designed 0D/2D contact interface yields abundant highly efficient active sites, thereby markedly enhancing the photocatalytic reduction activity of CO_2_.

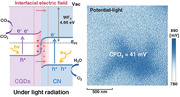

## 1 Introduction

Photocatalytic conversion of atmospheric CO_2_ into valuable fuels stands as a critical avenue for advancing global environmental and energy sustainability, being consistent with the overarching goal of carbon neutrality.^[^ ^1^, ^2^, ^3^ ^]^ Metal‐free carbon nitride (CN) polymers, distinguished by excellent thermal and chemical stability, facile production, and an appropriate bandgap, have emerged as promising photocatalysts for solar‐driven conversion.^[^ ^4^, ^5^ ^]^ Unfortunately, bulk CN suffers from inherent drawbacks to impede its photocatalytic efficacy, including low specific surface areas, suboptimal utilization of visible light, and pronounced carrier recombination.^[^ ^6^, ^7^ ^]^ Various strategies, including morphology modifications,^[^ ^8^ ^]^ heteroatom doping,^[^ ^4^, ^9^ ^]^ compounding,^[^ ^10^ ^]^ and defect construction,^[^ ^11^ ^]^ have been explored to overcome these challenges.

Morphological engineering, particularly the conversion from bulk to nanosheets, proves advantageous by reducing the transferring distance of carrier to the catalytic surface.^[^ ^12^ ^]^ Additionally, the construction of heterojunctions has demonstrated significant potential in facilitating charge separation within the spatial domain, thereby enhancing overall photocatalytic performance.^[^ ^13^, ^14^ ^]^ Crucially, the intrinsic challenges posed by the thermodynamic contradiction between light absorption and redox potential are effectively addressed by the built‐in electric field at the heterojunction interface, also known as the interfacial electric field.^[^ ^15^, ^16^ ^]^ This dynamic field proves pivotal in overcoming the aforementioned contradiction and expediting the efficient separation of charge carriers.^[^ ^17^, ^18^ ^]^ The selection of an appropriate anti‐semiconductor to construct a durable interface with CN becomes paramount for enhancing the interface electric field, thereby promoting charge transfer at the interface.^[^ ^19^ ^]^ In a surprising turn, zero‐dimension carbon quantum dots (CQDs), recognized as adept light capturers and electron receptors, emerge as innovative contributors with light‐capturing and electron‐receptor properties, featuring their unique optical property of tunable bandgaps.^[^ ^20^ ^]^ Therefore, CQDs have been integrated into the design of superior photocatalysts through successful coupling with CN semiconductors.^[^ ^21^, ^22^ ^]^ This integration results in an enhanced catalytic activity, achieved by impeding the recombination of photo‐excited carriers and broadening the absorption range of visible light.^[^ ^23^, ^24^ ^]^

In this study, we embarked on a comprehensive exploration of a high‐quality photocatalytic system, leveraging a design involving an enriched nitrogen‐doped CQDs. Then, employing a CQDs induced hydrothermal‐calcination tandem synthesis strategy, we successfully engineered a 0D/2D CQD/CN heterojunction. The resulting CQD/CN heterojunctions exhibit enhanced absorption of visible light, enriched carrier concentrations and efficient charge separation, accompanied by large surface area. collectively culminating in an excellent CO_2_ reduction rate. Mechanistic insights reveal that the internal electric field between CQDs and CN serves as a powerful driving force for the photoinduced electron transfer at the interface, facilitating the carrier transfer process and realizing the accumulation of highly reductive photoelectrons in the micro CQDs region. Our findings not only advance the development of efficient non‐metallic photocatalysts but also deepen the understanding of the underlying mechanisms governing their enhanced performance.

## 2 Results and Discussion

**Figure 1:**
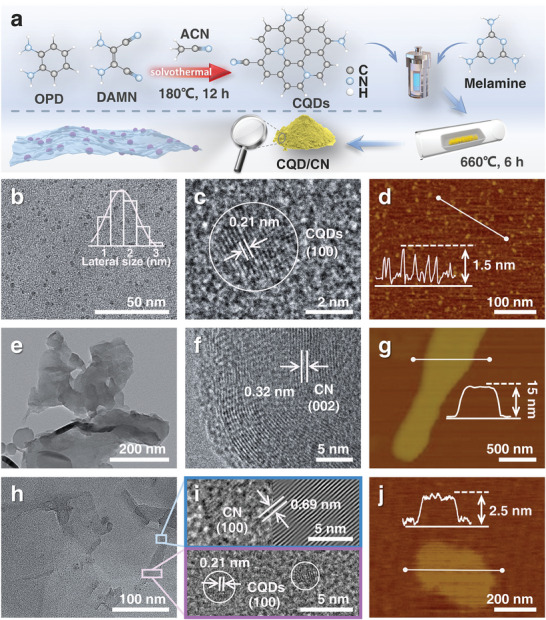
a) Schematic preparation process of CQD/CN. b) The TEM, c) HRTEM, and d) AFM images of CQDs. e) The TEM, f) HRTEM, and g) AFM images of CN. h) The TEM, i) HRTEM, and j) AFM images of CQD/CN.

The 0D/2D CQD/CN heterojunction involved a hydrothermal‐calcination tandem synthesis strategy induced by CQDs, as illustrated in **Figure** **1**a. The initial stage encompassed the preparation of nitrogen‐doped CQDs through a simple hydrothermal treatment utilizing o‐phenylenediamine (OPD) and diaminomalononitrile (DAMN) as precursors, with precise implementation of a surface charge‐state modulation strategy elucidated in the experimental section. Subsequent to the integration process commenced as the CQDs were introduced into the reactor, where thermal polymerization with melamine occurred under consistent hydrothermal conditions. The ensuing reaction intermediates underwent controlled calcination at 660 °C in an N_2_ environment for 6 h, yielding sample labeled as CQD/CN.

Transmission electron microscopy (TEM) was utilized to observe the morphological attributes of the CQDs (Figure 1b), CN (Figure 1e), and the synthesized 0D/2D CQD/CN heterojunction (Figure 1h). Importantly, these TEM images unveil the monodisperse nature of the CQDs, exhibiting a diameter close to 1.5 nm. Meanwhile, both CN and CQD/CN manifest themselves as thin sheets. A notable observation is the well‐dispersed arrangement of CQDs on their surface of CQD/CN, highlighting the successful integration of CQDs with CN. In‐depth analysis through high‐resolution TEM (HRTEM) reveals a crystallite plane spacing of 0.21 nm in CQDs (Figure 1c), being consistent with the characteristic (100) spacing of graphitic carbon.^[^ ^25^ ^]^ Furthermore, the discernible diffraction fringe of 0.32 nm (Figure 1f) corresponds to the (002) crystal face of CN, indicative of the interlayer stacking within the conjugate aromatic series.^[^ ^7^ ^]^ Moreover, TEM image of CQD/CN nanosheets (Figure 1i) displays diffraction fringes corresponding to the (100) crystal faces of both CQDs (0.21 nm) and CN (0.69 nm),^[^ ^26^, ^27^ ^]^ providing well‐defined evidence of the successful integration of CQDs into CN and the simultaneous formation of numerous tiny interface regions endowed with a rich interfacial electric field. In addition, energy‐dispersive X‐ray (EDX) mapping imaging (Figure S1, Supporting Information) corroborates the uniform distribution of C, N, and a trace O elements within the CQD/CN structure. To ascertain the thickness of these samples, atomic force microscopy (AFM) measurements were conducted. The determined height range of CQDs falls between 1.0 and 1.5 nm (Figure 1d), corresponding to the thickness of three to four graphene layers. In contrast, bulk CN exhibits a thickness of up to 15 nm (Figure 1g). Surprisingly, CQD/CN presents an unexpected thickness of 2.0 nm (Figure 1j). Further insights are garnered from TEM images of the precursor (Figure S2, Supporting Information), indicating that the incorporation of CQDs in precursor results in a thinner synthesized products compared to the controlled sample without CQDs. This observation suggests a pivotal role for CQDs in regulating the precursor size, ultimately leading to the formation of ultra‐thin CQD/CN nanosheets. The ultrathin nanosheet architecture of CQD/CN endows it with an expansive specific surface area, providing abundant photocatalysis active sites.

**Figure 2:**
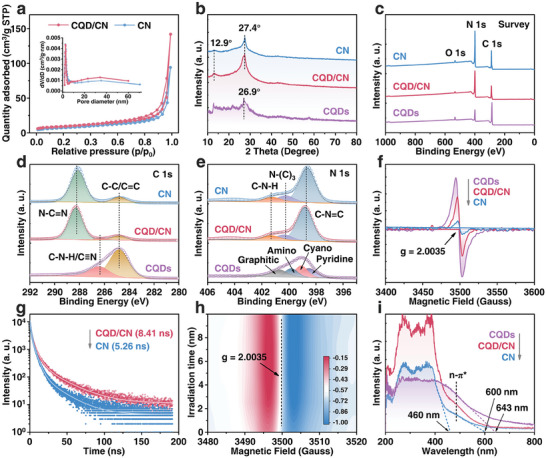
a) Nitrogen adsorption–desorption isotherms and the corresponding pore‐size distributions (inset) of CN and CQD/CN. b) The XRD patterns of CQDs, CN, and CQD/CN. The c) XPS Survey, d) XPS C 1s, and e) XPS N 1s spectra of CQDs, CN, and CQD/CN. f) The EPR spectra of CQDs, CN, and CQD/CN. g) The TR‐PL spectra of CN, and CQD/CN. h) The in situ EPR spectrum of CQD/CN. i) The DRS spectrum of CQDs, CN, and CQD/CN.

To validate the supposition, the N_2_ adsorption‐desorption measurements elucidating the pore structure and surface area of the materials are depicted in **Figure** **2**a. Both CN and CQD/CN exhibit type‐IV isotherms, as per the IUPAC categorization standard, accompanied by distinctive capillary condensation or evaporation steps, indicative of an orderly mesoporous structure.^[^ ^28^ ^]^ Notably, CQD/CN, identified as the mesoporous material, demonstrates a higher adsorption dV/dD pore volume and surface area than CN.^[^ ^29^ ^]^ This observation underscores the substantial increase in specific surface area of CQD/CN facilitated by the CQDs‐induced hydrothermal‐calcination tandem synthesis strategy. The existence of these porous structures can facilitate the adsorption of reactants, offering additional active sites for the photocatalysts, thus fortifying their photocatalysis capability.

Crystal structure modifications were investigated using X‐ray diffraction (XRD) spectroscopy (Figure 2b). Both CN and CQDs/CN exhibit two distinct characteristic peaks, namely a peak (100) at 12.9° and a peak (002) at 27.4°, representing tris‐triazine duplication and interlayer stacking, respectively.^[^ ^30^ ^]^ In the case of CQDs, a peak (002) appears at 26.9°, originating from the graphitic layer stack.^[^ ^31^ ^]^ Interestingly, the implantation of CQDs onto CN enhances the (002) diffraction peak strength of CQD/CN, signifying the effective integration of CQD and CN during the hydrothermal and calcination processes, attesting to the refined crystallinity and structural integrity achieved through this innovative synthesis approach.

The FTIR spectra, presented in Figure S3 (Supporting Information), provide insights into the molecular composition of CQDs, CN, and CQD/CN. For CN and CQD/CN, the peak at 810 cm^−1^ corresponds to the out‐of‐plane bending mode of heptazine rings, while the collective peaks between 1200 and 1600 cm^−1^ delineate the stretching and bending modes of the C─N conjugated heterocycles within the “melon” framework.^[^ ^32^ ^]^ Furthermore, the broad peaks in the 3000–3300 cm^−1^ range are correlated to amine group (N─H) tensile vibrations.^[^ ^7^ ^]^ Concurrently, the infrared signal at 2233 cm^−1^ in CQDs is assigned to the stretching vibration of the cyano group (C≡N).^[^ ^33^ ^]^

The component and element characterization of CQDs, CN, and CQD/CN was further conducted using XPS spectra. The results of the XPS survey (Figure 2c) affirm the predominant presence of C and N elements, with a trace of O elements (Table S1, Supporting Information), consistent with EDX mapping observations. In the C 1s spectra of CQD/CN (Figure 2d), the binding energies at 284.8, 286.2, and 288.3 eV were assigned to graphitic carbon (C─C/C═C),^[^ ^34^ ^]^ amino or cyano group (C─N─H/C≡N),^[^ ^33^ ^]^ and sp^2^‐hybridized carbon (N─C═N),^[^ ^5^ ^]^ respectively. The N1s spectra (Figure 2e) of CN and CQD/CN revealed three peaks corresponding to sp^2^‐hybridized N (N─C═N) (398.8 eV), sp^3^‐hybridized N (N─(C)_3_) (400.4 eV), and amine functional groups (C─N─H) (401.3 eV), consistent with the heptazine‐based architecture.^[^ ^4^ ^]^ In addition, the N 1s spectrum of CQDs could be fitted with pyridine N (398.5 eV), cyano N (399.1 eV), amino N (399.8 eV), and graphitic N (400.7 eV).^[^ ^35^, ^36^, ^37^ ^]^ Moreover, weak XPS O 1s spectra (Figure S4, Supporting Information) in all samples indicate oxygen contamination in the environment.^[^ ^21^ ^]^ The combined XPS and FT‐IR results indicate the various forms of nitrogen elements in CQDs, including graphitic N, amino N, cyano N, and pyridine N. The substantial N element doping (23.43%) enriches CQDs with electrons, effectively improving the π delocalized electron cloud density of CQDs.

The electronic properties of the catalyst were characterized using EPR, as depicted in Figure 2f.^[^ ^38^ ^]^ All catalysts exhibited a distinct EPR signal peak at g = 2.0035, proving the presence of de‐localized electrons.^[^ ^39^ ^]^ Notably, CQDs exhibited the strongest EPR peaks, indicating the abundance of π delocalized electrons in N‐doped CQDs. Remarkably, the EPR signal of CQD/CN was ≈4.3 times higher than that of CN, attributing this enhancement to the ultrathin nanosheet structure of CQD/CN, which exposes more delocalized electrons. The integration of CQDs with CN results in an expanded π‐conjugate system. Therefore, CQDs embedded on the surface of CN disperse numerous “high charge density points”, inducing a rich micro‐region interface electric field. This naturally enhances the photoelectrochemical characteristics and catalytic activity of CQD/CN.

A series of optical and electrochemical characterizations were employed to monitor the charge separation and transfer kinetics of photocatalysts. Under visible light radiation (λ > 420 nm), CQD/CN exhibited a higher transient photocurrent compared to CN (Figure S5, Supporting Information), indicating superior visible‐light responsiveness and improved efficiency of charge separation in CQD/CN. The charge transfer resistance (Rct) of CQD/CN showed a significant reduction compared to CN, implying higher electron exchange efficiency on the surface of CQD/CN, as evidenced by the smaller EIS radius (Figure S6, Supporting Information).^[^ ^40^ ^]^ Moreover, the fluorescence intensity of CQD/CN was notably lower than that of CN (Figure S7, Supporting Information), signifying that the introduction of CQDs into CN effectively inhibits the recombination of photogenerated electron–hole pairs.^[^ ^41^ ^]^ Additionally, the decay kinetics of photogenerated carriers were predicted by fitting the time profiles of normalized transient absorption in the range of 365–1000 nm (Figure 2g; Table S2, Supporting Information). Compared to the average lifetime of pure CN (5.26 ns), the average lifetime of CQD/CN was measured at 8.41 ns, indicating efficient charge transfer and suppressed electron–hole recombination in the heterojunction.^[^ ^42^ ^]^ In situ EPR (Figure 2h) was employed to analyze the change of charge density of the CQD/CN heterojunction after illumination.^[^ ^43^ ^]^ The EPR signal of CQD/CN powder gradually increased as light radiation commenced, reached stability at 7 min. This indicates that intense electron‐hole pair separation occurred in the catalyst after the photoexcitation, resulting in the gradual accumulation of unpaired electron concentration, maintaining dynamic equilibrium after reaching the maximum amount.

The light absorption properties of the synthesized samples were investigated using UV–vis diffuse reflection spectroscopy (DRS, Figure 2i). In particular, the prepared CN displayed a distinct absorption edge at ≈600 nm, showcasing a significant redshift compared to the intrinsic absorption edge of classical graphite‐phase CN (≈460 nm).^[^ ^44^ ^]^ This redshift suggests the activation of the n–π* transition through the hydrothermal calcination synthesis strategy.^[^ ^45^ ^]^ On the other hand, CQDs exhibited noticeable absorption across the entire visible region, indicative of their narrow bandgap. The integration of CQDs with CN in the heterojunction, denoted as CQD/CN, led to a substantial enhancement in visible light absorption, which is a favorable aspect for improving photocatalytic performance. Moreover, based on the Kubelka–Munk expression (Figure S8, Supporting Information), the calculated indirect bandgap values for CN and CQDs were determined to be 2.50 and 2.40 eV, respectively.^[^ ^46^ ^]^

Understanding the energy band structures of semiconductors is crucial for delineating their oxidation‐reduction capabilities and ultimately determining their charge separation efficiency. Mott–Schottky curves at different frequencies were carried out to clarify the conductive type and flat‐band potential of the aforementioned samples (Figure S9, Supporting Information).^[^ ^47^ ^]^ Both CN and CQDs exhibited linear graphs with positive slopes, indicative of typical n‐type semiconducting behavior and suggesting excellent electron conduction properties.^[^ ^48^ ^]^ Besides, the flat band potential of CN and CQDs was determined to be ≈−0.31 and −0.57 V (vs Ag/AgCl, pH 7), respectively. According to the band structure relationship, the conduction band/valence band (CB/VB) of CN and CQDs were confirmed as −0.31/1.33 V and −0.57/0.75 V (vs NHE, pH 7), respectively (Figure S10, Supporting Information).

**Figure 3:**
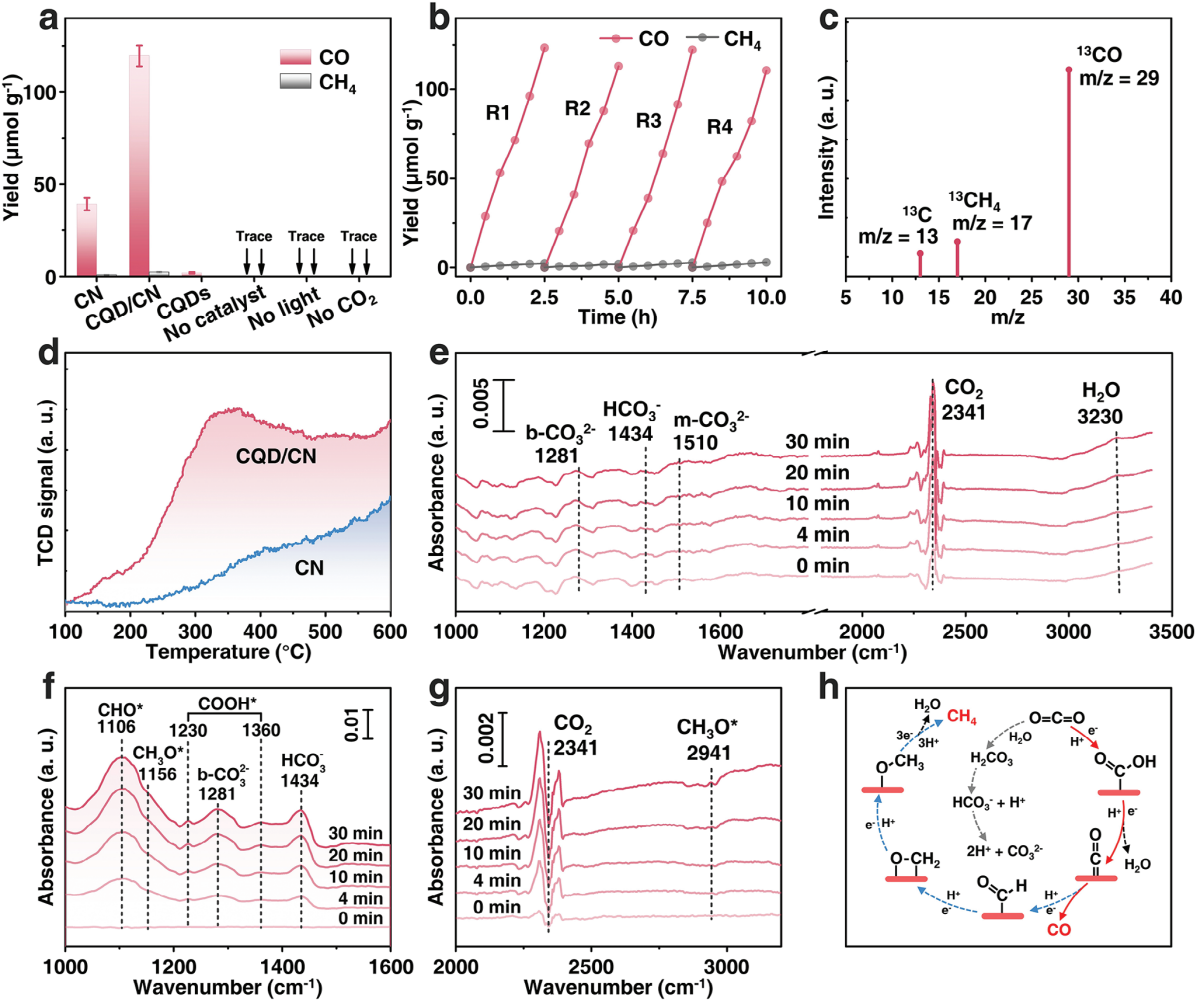
a) The CO_2_ photoreduction activities of the synthesized samples as well as control experiments. b) The time course of the photocatalytic activity of CQD/CN. c) Mass spectrum of the products from the ^13^CO_2_ photoreduction by CQD/CN. (d) CO_2_‐TPD profiles of CN, and CQD/CN. In situ DRIFTS profiles of CQD/CN for e) dark and f,g) illumination processes. h) Possible mechanism pathways of CO_2_ reduction to CO and CH_4_.

The photoreduction activity of the catalyst was systematically assessed in a gas‐solid reaction system under visible light irradiation (λ > 420 nm). Control experiments (**Figure** **3**a) were conducted without catalyst, light, or CO_2_. The results demonstrated the absence of reduction products under these conditions, affirming the indispensability of both light and catalyst in the photocatalytic CO_2_ reduction process. Importantly, it was confirmed that the reduction products originated from CO_2_ rather than the self‐decomposition of the catalyst. CO emerged as the primary reduction product, accompanied by a minor amount of methane (Figure S11, Supporting Information). Following 2.5 h of visible light exposure, pure CN and CQDs exhibited relatively low CO production, measuring 39.2 and 2.0 µmol g^−1^, respectively. Impressively, the photocatalytic activity of CQD/CN surpassed that of pure CN, achieving an average CO production rate of 120 µmol g^−1^, outperforming most other semiconductor photocatalytic materials reported for similar environments (Table S3, Supporting Information). In addition, CQD/CN demonstrated high CO selectivity (92.8%) and exhibited superior operational stability, with no significant inactivation occurring over 10 h of continuous measurements (Figure 3b). To further verify the origin of the product, isotopically labeled ^13^CO_2_ experiments were carried out by mass spectrometry (Figure 3c). The results revealed a distinct and robust signal of ^13^CO at m/z = 29, confirming that the photocatalytic product CO originates from ^13^CO_2_ rather than the catalyst itself.

The CO_2_‐temperature‐programmed desorption (CO_2_‐TPD, Figure 3d) analysis was employed to explore the chemical adsorption capacity of the catalyst for CO_2_. The results revealed a distinct oxygen desorption peak at 350 °C for CQD/CN, proving an abundance of basic sites with moderate strength on the catalyst surface.^[^ ^49^ ^]^ This characteristic not only facilitates the adsorption of CO_2_ molecules but also aids in the desorption of photocatalytic products. Moreover, the strength of oxygen desorption peak in CQD/CN is significantly higher than that of pure CN. This is attributed to the ultra‐thin morphology of CQD/CN, exposing more alkaline sites compared to CN, thereby contributing to superior photocatalytic CO_2_ reduction activity.

Evolving species during photocatalyzed CO_2_ reduction process by CQD/CN were monitored by in situ diffuse infrared Fourier transform spectroscopy (DRIFTS). Positive peaks in the infrared spectrum were assigned to the accumulation of different intermediate species. In the initial 30 min of the dark reaction (Figure 3e), CQD/CN was exposed to a flowing CO_2_ and H_2_O gas environment to ensure adsorption saturation. The gradual enhancement of CO_2_ (2341 cm^−1^) and H_2_O (3230 cm^−1^) vibration signals on the surface of CQD/CN,^[^ ^12^, ^50^ ^]^ along with the emergence of multiple carbonate species, including m‐CO_3_2^−^ (1510 cm^−1^), b‐CO_3_2^−^ (1281 cm^−1^), and HCO_3_^−^ (1434 cm^−1^), validates the effective adsorption and activation of raw material molecules by CQD/CN.^[^ ^8^ ^]^

In addition, the evolution of species adsorbed on the surface of CQD/CN during light irradiation was tracked (Figure 3f,g). The negative increase in the CO_2_ signal indicates the consumption of CO_2_ as a raw material in the photoreaction system. Peaks at 1230 and 1360 cm^−1^ were attributed to COOH*,^[^ ^51^ ^]^ a crucial intermediate species for converting CO_2_ to CO or CH_4_. Furthermore, the peaks at 1097 cm^−1^ belong to CHO*, and the peaks at 1158 and 2941 cm^−1^ belong to CH_3_O*, which are important intermediates in the formation of CH_4_.^[^ ^52^, ^53^ ^]^ The evolution of these intermediates in DRIFTS corroborates the effective photocatalytic reduction of CO_2_ by CQD/CN. According to the above results, the CO_2_ conversion process in the photocatalytic system can be summarized (Figure 3h). Initially, CO_2_ molecules adsorbed on the surface of CQD/CN are reduced to COOH* by photoelectrons, subsequently obtaining e^−^ and protons to form CO. Moreover, a fraction of adsorbed CO* undergoes successive reduction to CHO*, and CH_3_O*, eventually resulting in CH_4_ formation.^[^ ^54^ ^]^

**Figure 4:**
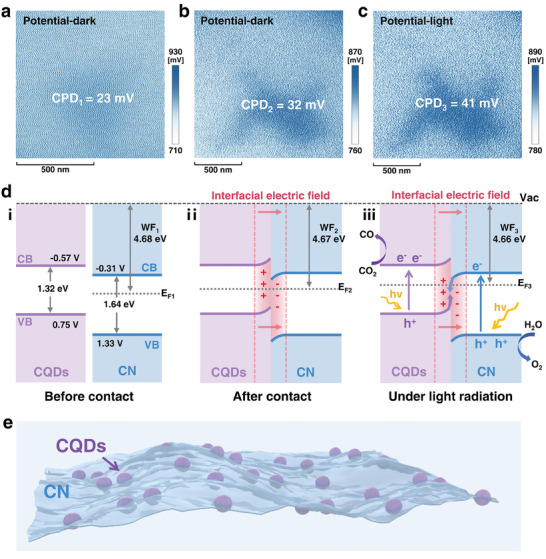
CPD of a) CN in dark, b) CQD/CN in dark, and c) CQD/CN in light detected with in situ KPFM. d) Schematic of the built‐in electric field to facilitate efficient charge transfer process. e) Schematic diagram of the morphology of CQD/CN.

To monitor the charge transfer dynamics within CQD/CN heterojunction, in situ Kelvin probe force microscopy (KPFM) was employed to record the surface potential of samples under dark and illumination conditions.^[^ ^55^ ^]^ The results illustrated that in the dark, the contact potential difference (CPD) between CN and the probe (**Figure** **4**a, CPD_1_ = 23 mV) was lower than the CPD between CQD/CN and the probe (Figure 4b, CPD_2_ = 32 mV). This discrepancy implied an enhancement in surface charge density following the integration of CQDs. Importantly, as the environment transitioned from darkness to light, the CPD between CQD/CN and the probe (Figure 4c, CPD_3_ = 41 mV) significantly exceeded CPD_2_ by a notable margin. This observation suggested a heightened charge transfer between CN and CQDs in CQD/CN when exposed to light, facilitating the accumulation of charge on the surface.

The nuanced interfacial charge transfer mechanisms between CN and CQDs were further elucidated through the calculation of the work function (WF) of the samples according to the formula WF (eV) = WF_probe_ + e × CPD (Figure S12, Supporting Information, for details).^[^ ^56^ ^]^ After determining the WF value, it became evident that the Fermi level (E_F_) of CQD/CN was higher than that of pure CN (Figure 4d), signifying the spontaneous transfer of electrons from CQDs to CN through the heterogeneous interface when CN and CQDs are in close contact. This transfer causes the E_F_ of CQDs and CN to move down and up, respectively, until their E_F_ reaches equilibrium.^[^ ^57^ ^]^ This transfer induced the generation of an interfacial electric field from CQDs to CN, manifesting in the bending of energy bands.^[^ ^58^ ^]^ This interfacial electric field acted as a robust driving force, orchestrating the swift combination of photogenerated electrons at CB of CN with the holes at VB of CQDs.^[^ ^59^ ^]^ This intricate interplay resulted in the directed accumulation of reduced photoelectrons in the micro‐region of CQDs, finely tuned by the interfacial electric field, ultimately driving efficient CO_2_ reduction to CO and CH_4_ (Figure 4e).

**Figure 5:**
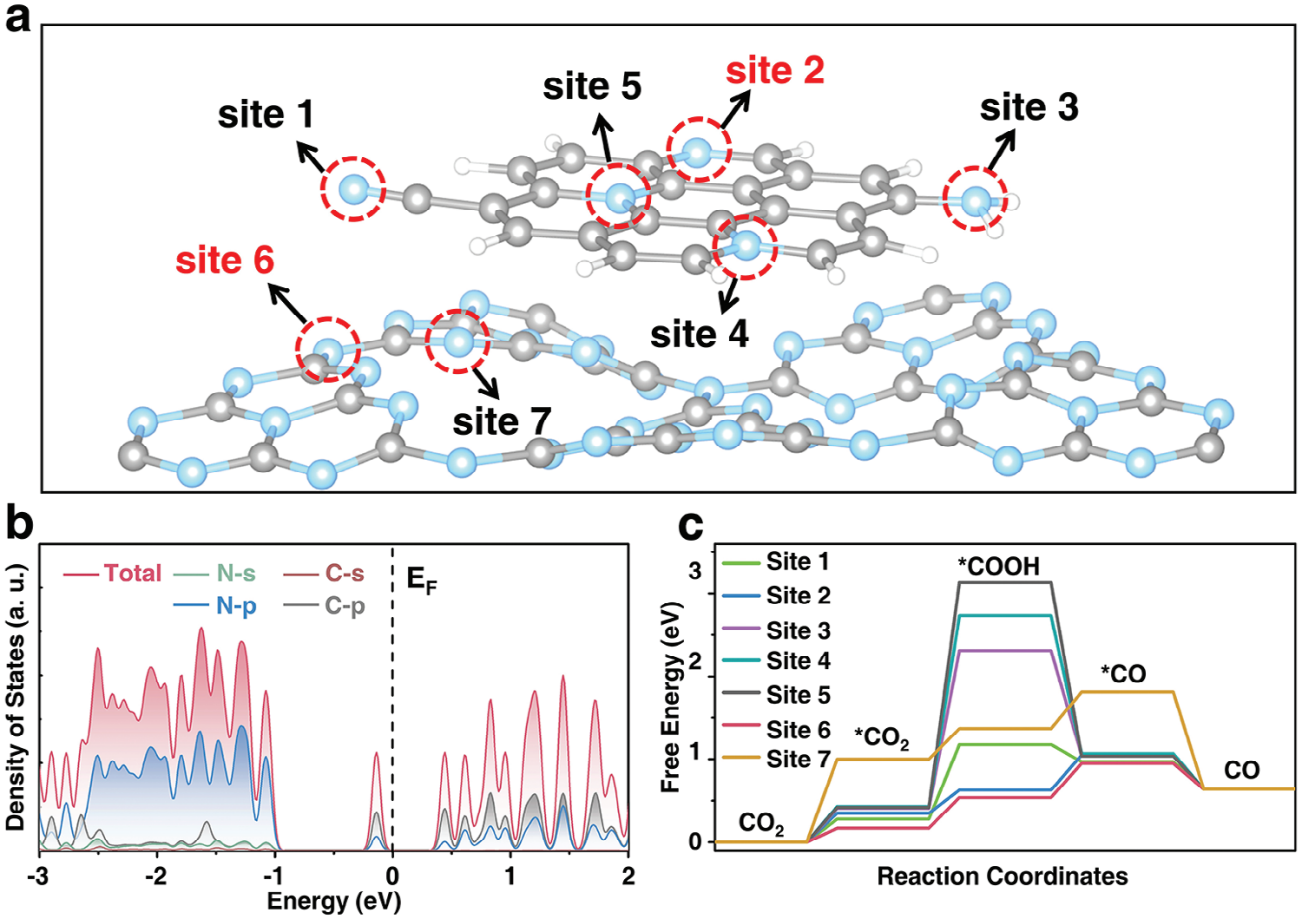
a) Optimized unit cells and b) calculated density of states for CQD/CN. c) Gibbs free energies of CO_2_ photoreduction pathways by DFT calculations over possible active sites on the surface of CQD/CN.

Density functional theory (DFT) calculations were carried out to elucidate the impact of introduced CQDs on the charge dynamics of CN.^[^ ^60^ ^]^ Post‐structural optimization, the calculated bandgap values for CQD, CN, and CQD/CN were determined to be 0.36, 1.35 (Figure S13, Supporting Information), and 0.34 eV (**Figure** **5**a,b), respectively. These findings indicate that the integration of CQDs into CN results in a narrower bandgap in CQD/CN. In intricate detail, the interaction between the carbon ring and heptazine unit generates multiple intermediate energy levels above the VB, establishing a novel transmission path for long‐wavelength visible light.^[^ ^6^ ^]^ In addition, the Gibbs free energy of the reaction unveils the active site of CO2 photoreduction on samples (Figure S14, Supporting Information; Figure 5c). From a thermodynamic standpoint, it is conventionally recognized that COOH* formation represents a rate‐limiting step in the CO_2_ reduction process.^[^ ^51^ ^]^ Calculations for each nitrogen site in the CQD/CN model reveal that bridging nitrogen on CN at the interface (site 6) and pyridine nitrogen at the edge of CQDs (site 2) exhibit low COOH* generation energy. This indicates that embedding CN within CQDs introduces abundant active sites at the interface, consequently enhancing CO production.

## 3 Conclusion

In summary, the fabrication of 0D/2D CQD/CN heterojunction through a nitrogen‐rich CQDs‐induced hydrothermal‐calcination tandem synthesis strategy represents a cost‐effective and innovative approach. This method ensures the formation of a heterojunction with reduced thickness, amplified surface area, and heightened crystallinity. The integration of CQDs induces structural reconfiguration in CN, leading to the exposure of abundant built‐in electric fields on the surface. This structural transformation also imparts superior optical characteristics to CQD/CN, characterized by attenuated charge recombination, and heightened electron exchange efficiency, which is beneficial for accelerating the photocatalysis reduction process. Remarkably, the CQD/CN heterojunction demonstrates a substantial enhancement in photocatalytic CO_2_ reduction performance, yielding an average CO production rate of 48 µmol g^−1^ h^−1^, a threefold increase compared to pure CN. This profound understanding of charge transfer dynamics within the CQD/CN heterojunction advances fundamental insights into complex photocatalytic processes. Moreover, the strategic integration of CQDs offers a versatile platform for advancing the field of semiconductor photocatalysis, underscoring the potential for broader implications in sustainable energy technologies.

## Conflict of Interest

The authors declare no conflict of interest.

## Supporting information

Supporting Information

## Data Availability

The data that support the findings of this study are available from the corresponding author upon reasonable request.
