# Supplementary material for: Engineering Built‐In Electric Field Microenvironment of CQDs/g‐C3N4 Heterojunction for Efficient Photocatalytic CO2 Reduction
Source: Adv Sci (Weinh). 2024 May 10;11(28):2403607. doi: 10.1002/advs.202403607 (PMC11267297; doi:10.1002/advs.202403607)
Supplement: Supplementary file 1 — Supporting Information [file ADVS-11-2403607-s001.docx]

Supporting Information

Engineering Built-In Electric Field Microenvironment of CQDs/g-C_3_N_4_ Heterojunction for Efficient Photocatalytic CO_2_ Reduction

Yun Xu^†^, Weidong Hou^†^, Kai Huang, Huazhang Guo, Zeming Wang, Cheng Lian, Jiye Zhang, Deli Wu, Zhendong Lei*, Zheng Liu* and Liang Wang*

***Experimental***

*Chemicals:* Acetonitrile (ACN) (99%^+^, Adamas), 2,3-diaminomaleonitrile (DAMN) (98%+, Adamas), Melamine (99%, Adamas), o-Phenylenediamine (OPD) (99%, Adamas). All chemicals were used without any further purification.

*Synthesis of CQDs sample:* Dissolve 50 mg of OPD and 25 mg of DAMN in 50 mL of ACN. Transfer to a 100 ml reactor and heat at 180 °C for 12 hours. After cooling to room temperature, the reaction mixture was filtered (0.22 µm), rotary evaporated, dialyzed, and freeze-dried to obtain solid CQDs.

*Synthesis of CN sample:* The 5 g melamine were dispersed in 50 mL H_2_O and transferred to a 100 mL reactor for reaction at 180 ℃ for 12 h. A solid precursor is obtained. The precursor was then annealed in a tube furnace at 660 °C for 6 h at a heating rate of 2.5 °C·min^-1^ in an N_2_ atmosphere. Finally, the **CN** solid powder is obtained.

*Synthesis of* *CQD/CN sample:* The 5 g melamine and 20 mg CQDs were dispersed in 50 mL H_2_O and transferred to a 100 mL reactor for reaction at 180 ℃ for 12 h. A solid precursor is obtained. The precursor was then annealed in a tube furnace at 660 °C for 6 h at a heating rate of 2.5 °C·min^-1^ in an N_2_ atmosphere. Finally, the **CQD/CN** solid powder is obtained.

***Photocatalytic CO_2_ reduction tests***

The reaction system adopts the gas-solid mode.^[1]^ First, 5 mg catalyst was dispersed in 5 mL deionized water for 20 min by ultrasonic to obtain a suspension. The suspension was then applied to a 7 cm diameter round glass sheet with a single frosted surface (coated on the frosted side) and vacuum-dried at 60°C. Afterward, the glass sheet was placed above the inside of the 780 ml double-layer reaction cell that contained 30 ml DI H_2_O. The glass sheet was supported by a glass tube. Before illumination, N_2_ gas was introduced for 30 min to expel the air inside the reaction cell, and then the gas was converted to high-purity CO_2_ and purged for 20 min (flow rate: 115 mL/min). The light source is a 300 W xenon lamp (Perfectlight, PLS-SXE300). Driven by the micro air pump (flow rate about 1 L/min), the gas in the reaction tank circulates. And the reaction cell was kept at 25°C. The photocatalytic CO_2_ reduction gas products were detected by gas chromatography (GC-2060, Shanghai Ruimin Instrument Co., Ltd.) with flame ionization detector (FID). Each catalyst was tested three times in parallel to reduce chance factors.

***Photocatalytic stability***

After the above photocatalytic test, the DI H_2_O in the reaction tank was renewed, and 30 min N_2_ and 20 min CO_2_ were re-injected in turn for the next round of photoreaction. Repeat the same process four times to evaluate the stability of the catalyst.

***Control experiment***

Control experiments were conducted in the absence of light, catalyst, or N_2_ in place of CO_2_.

***Electrochemical measurements***

The 0.5 M Na_2_SO_4_ solution was used as the electrolyte solution (pH = 7). The 5 mg of the catalyst was dispersed in 1 mL solution (700 µL H_2_O, 250 µL isopropyl alcohol, and 50 µL Nafion PFSA Polymer), and then 100 µL mixture was uniformly spread on indium-doped tin oxide (ITO) glass (1 × 2 cm) to dry naturally, and then a working electrode was obtained. In the electrochemical characterization test, Ag/AgCl was used as a reference electrode, and graphite rod was used as an auxiliary electrode. Mott-Schottky test was performed with the potentials ranged from -0.6 to 0.6 V at selected frequencies of 300, 500, and 1000 Hz. A 50-watt xenon lamp (420 nm filter) was used as the visible light source for the photocurrent test. After the open circuit potential stabilized, init E entered the open circuit potential value and ran for 400 sec with a 60-sec cycle (30-sec light and 30-sec darkness). Electrochemical impedance spectroscopy was tested at init E 0 V, high frequency 100000 Hz, low frequency 1 Hz, and quiet time 2 sec.

***Characterizations***

The nickel-filtered Cu-Kα radiation source (XRD: Rigaku D/max-2500, Japan) were be employed to investigated the crystal phase structures of samples. The transmission electron microscopy (TEM: JEM-2100F, Japan) and the high-resolution TEM (HRTEM: JEOL JSM-6490, Japan) were used together to with mapping to observe microstructure characteristics, including morphology and element distribution, of photocatalysts. Atomic force microscopy (AFM) and Kelvin Probe Force Microscope (KPFM) images of catalysts were recorded by a Bruker Dimension ICON-XR. The diffuse reflectance infrared Fourier transform spectroscopy (DRIFTS) spectra and Fourier transform infrared (FT-IR) spectra were measured by a spectrometer (Thermofisher, IS50). Electron spin resonance (ESR) spectrum was detected by BRUKER EMXplus. X-ray photoelectron spectroscopy (XPS, Thermofisher, ESCALAB250Xi) with a monochromatic Al-Kα X-ray source was used to analyze the composition, abundance, and elemental binding energy of materials. UV-vis diffuse reflectance spectroscopy (DRS) was recorded on TU-1950 using BaSO_4_ as reflection standard. The photoluminescence (PL) spectroscopy were obtained by scanning a UV-vis spectrophotometer (Duetta, Horiba Scientific) at an excitation wavelength of 365 nm. Time-resolved photoluminescence spectra were collected on DeltaFlex (Horiba Scientific) modular fluorescence lifetime system. The temperature-programmed desorption (TPD) tests were conducted utilizing a Micromeritics Anton-Paar ChemBET characterization system with a quartz U-tube reactor and detected using a TCD. The surface areas and pore size parameters of samples were measured through Brunauer-Emmett-Teller (BET) analysis using an ASAP 2460 nitrogen adsorption analyzer (Micromeritics). Electrochemical impedance spectroscopy (EIS), Photocurrent spectra (i-t) and Mott-Schottky curves were recorded by electrochemical workstation CHI760E. The surface water contact angle was measured on an Angle Contact Metering System (JCY-1).

***Calculation for selectivity of CO***

On an electronic basis (8 e^-^ for the formation of CH_4_ and 2 e^-^ for CO), the reduction selectivity of CO was calculated using the following formula, where n represents the yield of CO_2_ photoreduction products (µmol) after 3 hours.

$\text{Selectivity of CO (\%) }\text{= }\frac{\text{2 × n}\text{CO}}{\text{2 × n}\text{CO }\text{+ 8 × n}\text{CH4}}\text{ }\text{× }\text{100\%}$ (1)

***Density functional theory calculations***

DFT calculations were performed in the Vienna ab initio Simulation Package (VASP)^[2]^ using projector augmented ware (PAW) pseudopotential.^[3, 4]^ The Perdew-Burke-Ernzerhof (PBE) method was used to establish the exchange correlation functional.^[5]^ The dispersion correction was described by the Grimme’s D3 parametrization.^[6]^ The energy cutoff was set to 500 eV. The Monkhorst-Pack k-point grid of 2 × 3 × 1 was constructed for the systems of CQD and CQD/CN, and the 2 × 2 × 1 Monkhorst-Pack k-point grid was constructed for the systems of CN. The configuration optimization were relaxed until the forces and the energy tolerances less than 0.02 eV/Å and 10^-5^ eV/atom, respectively. For three materials, the two-electron reaction path for CO_2_RR was considered in this work, which involves the following two paths:

$*+CO_{2}+H^{+}+e^{-}=*COOH$(Δ*G*_1_) (2)

$*COOH+H^{+}+e^{-}=*CO+H_{2}O$(Δ*G*_2_) (3)

where * means the surface of three materials. The change of Gibbs free energy (∆*G*) for each elementary reaction was calculated by the computational hydrogen electrode (CHE) model, which was proposed by Nørskov et al.^[7]^ The Gibbs free energies of each reaction step can be calculated by:

$G=E_{Total}+E_{ZPE}+\int C_{\text{p}}\text{ }dT-TS$ (4)

where *E_Total_* is the total energy of the system, *E_ZPE_* is the correction of zero-point energy. The$\int C_{p}\text{ }dT$can be calculated by vaspkit software.^[8]^ *TS* is the vibrational entropy of the adsorbed intermediate at room temperature (*T* = 298.15 K).

**Figure S1**. (a) High-angle annular dark-field scanning TEM image of CN/CQD and the corresponding energy-dispersive X-ray (EDX) mapping images of (b) C, (c) N, and (d) O elements. (e) EDX of CN/CQD.


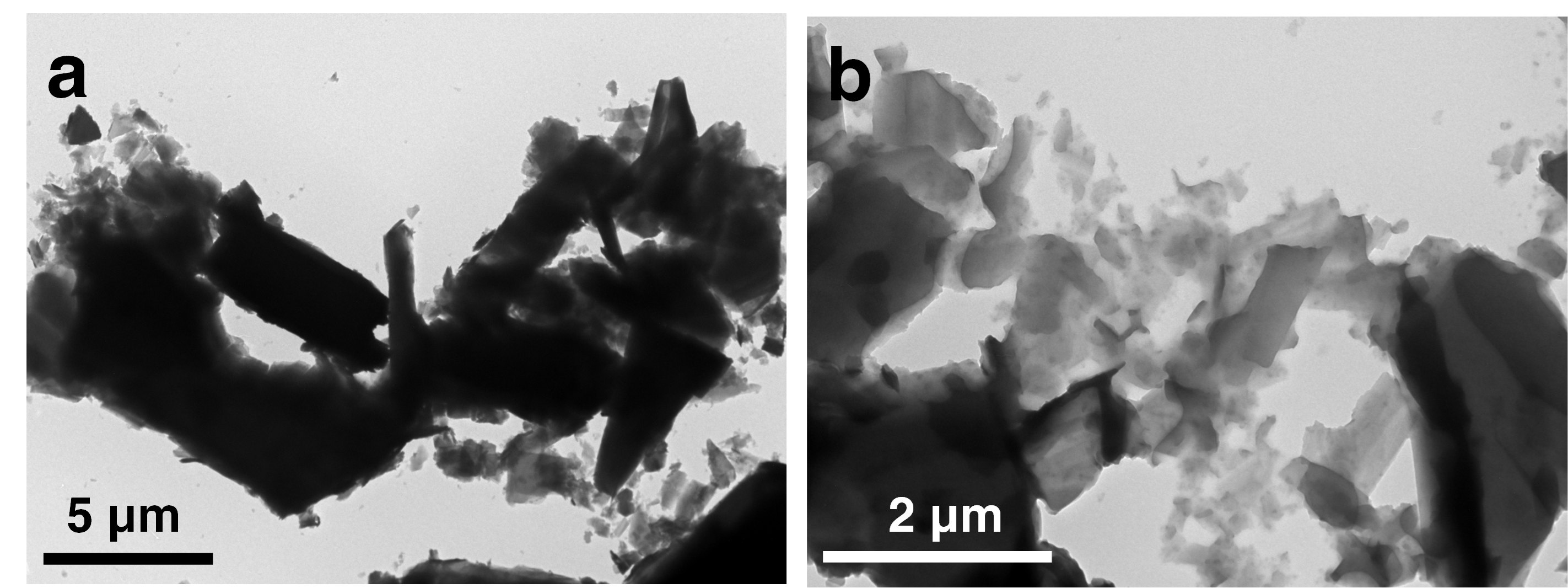


**Figure S2.** (a) TEM morphology of the precursor obtained by hydrothermal treatment of 5 g melamine. (b) TEM morphology of the precursor obtained by hydrothermal 5 g melamine and 20 mg CQDs together.

**Figure S3.** The FT-IR spectra of CQDs, CN, and CQD/CN.

**Figure S4.** The XPS Survey spectra of CQDs, CN, and CQD/CN.

**Figure S5.** The photocurrent curves of CN and CQD/CN.

**Figure S6.** The EIS spectra of CN and CQD/CN.

**Figure S7.** The PL spectra of CN and CQD/CN.

**Figure S8.** Transformed Kubelka-Munk functions vs. photon energy of CN and CQDs.

According to the Kubelka-Munk expression, the band gap can be calculated (**Figure S8**).

(*αhν*) = A(*hν*- *E*_g_)^n/2^  (5)

where α is the absorption coefficient, *h* is the Planck constant, *ν* is the optical frequency, and *E*_g_ is the band gap energy, A is the proportionality constant, and n is 4 owning to CN and CQDs are indirect transition semiconductor. Therefore, the band gap (*E*_g_) of CN and CQDs are 1.64 and 1.32 eV, respectively.

**Figure S9.** Mott−Schottky plots of CN (a) and CQDs (b) in 0.5 M Na_2_SO_4_ at pH = 7.

The Mott-Schottky plots of CN and CQDs were shown in **Figure S9**. The flat band potential of CN and CQDs are approximately -0.31 V and -0.57 V (vs. Ag/AgCl, pH = 7), respectively. For n-type semiconductor, the CB position usually more negative 0.1-0.3 eV than the flat band potential (0.2 eV used in this paper). In addition, the potential (vs. Ag/AgCl, pH = 7) can be converted to the normal hydrogen electrode (NHE) according to the formula:

*E*_NHE_ = *E*_Ag/AgCl_ + *E*^0^_Ag/AgCl_ (6)

where *E*^0^_Ag/AgCl_ = 0.197 V. Therefore, the CB of CN and CQDs are about -0.31 and -0.57 V (vs. NHE, pH = 7), respectively. In addition, according to the formula:

*E*_CB_ = *E*_VB_ - *E*_g_ (7)

The VB of CN and CQDs can be confirmed as 1.33 and 0.75 V (vs. NHE, pH = 7), respectively.

**Figure S10.** band structure of CN and CQDs.

**Figure S11.** GC (GC-2060, Shanghai Ruimin Instrument Co., Ltd.) raw data: (a) Gas chromatography spectra of CO and CH_4_ as photocatalytic CO_2_ reduction products. (b) Gas chromatographic spectra of photocatalyzed oxidation product O_2_ and reaction substrate CO_2_.


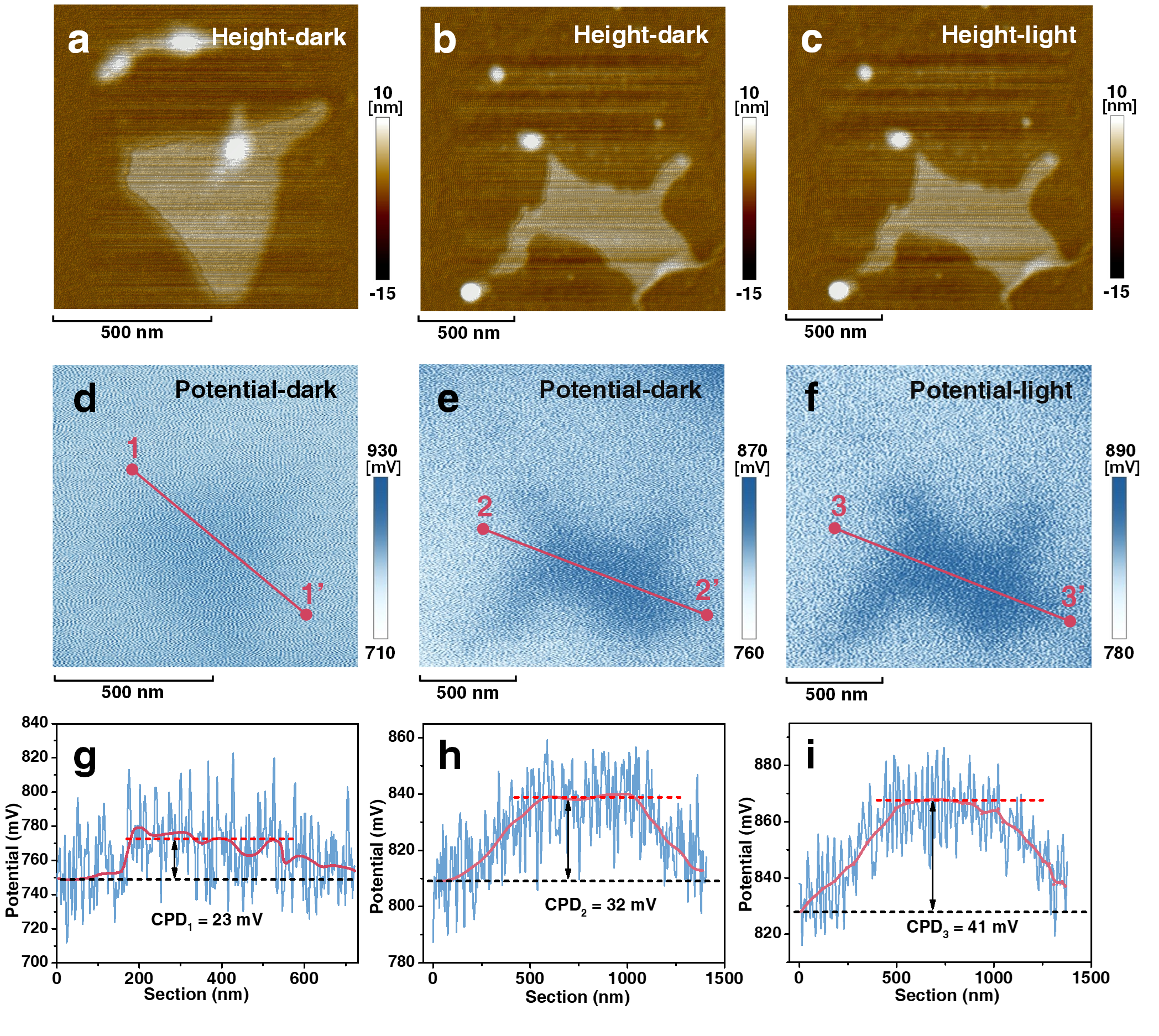


**Figure S12.** (a) Atomic force microscope (AFM) image in dark conditions of CN. AFM images in (b) dark and (c) light conditions of CQD/CN. (d) Kelvin Probe Force Microscope (KPFM) image in dark conditions of CN. KPFM images in (e) dark and (f) light conditions of CQD/CN. The liner scanning of contact potential differences (CPD) for samples along the (g) 1-1’, (h) 2-2’, and (i) 3-3’ lines.

**Figure S12** shows the measured value of the contact potential difference (CPD) by in-situ Kelvin Probe Force Microscope (KPFM) using gold flake as the reference. The CPD of CN in dark conditions is 0.023 V. And, the CPD of CQD/CN in dark and light conditions are 0.032 and 0.041 V, respectively. The work functions (WF) can be calculated according to the formula WF (eV) = 4.7 eV + e × CPD, where e is the charge of an electron and 4.7 eV is the work function of the corrected probe (SCM-PIT-V2). Therefore the WFs of CN in dark, CQD/CN in dark and CQD/CN in light can be determined to be 4.68, 4.67 and 4.66 eV.

**Figure S13.** Calculated density of states for (a) CQDs and (b) CN.


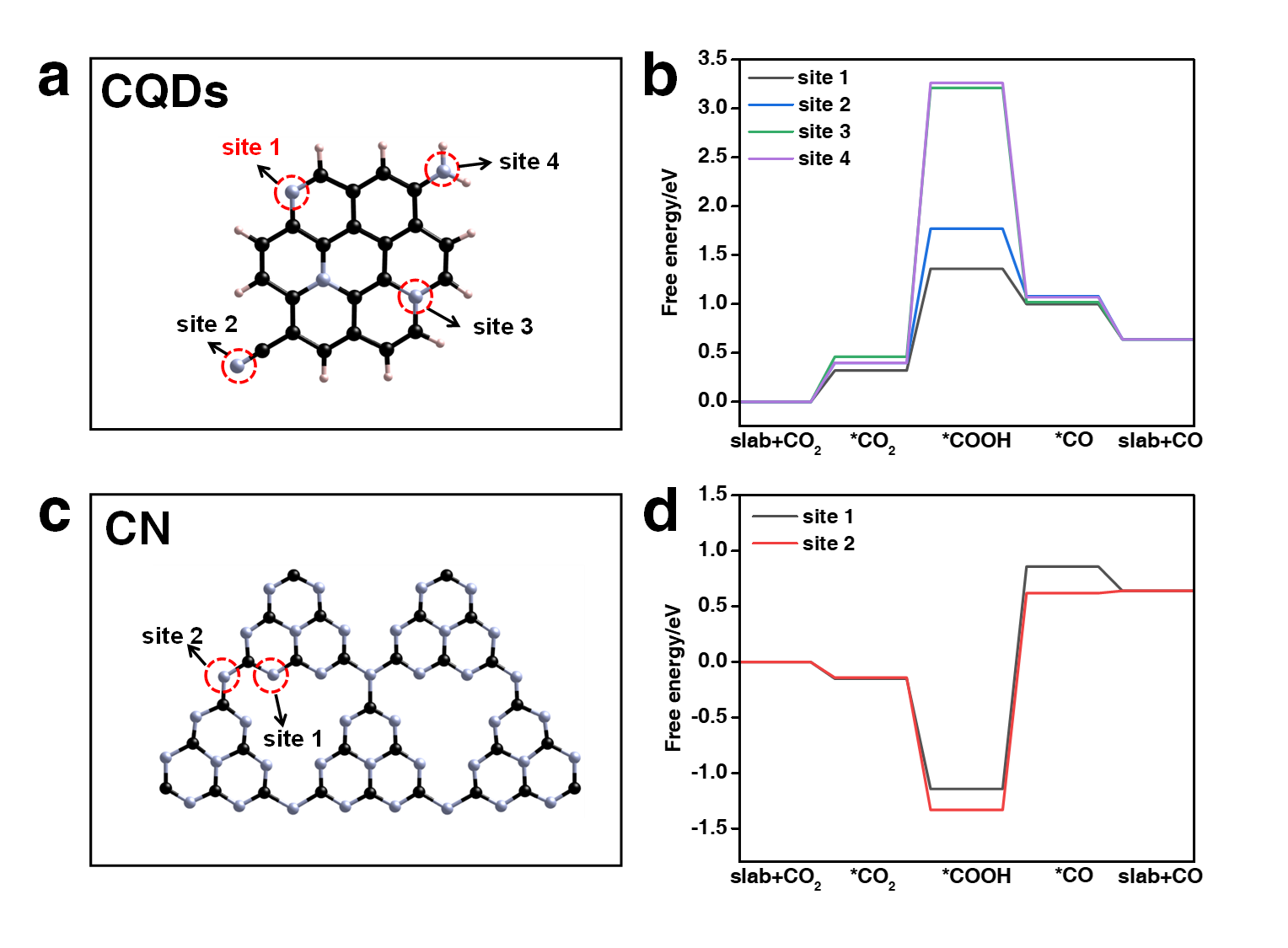


**Figure S14**. Optimized unit cells of (a) CQDs, and (c) CN. Gibbs free energies of CO_2_ photoreduction pathways by DFT calculations over possible active sites on the surface of (b) CQDs, and (d) CN.

**Table S1.** Surface compositions of C, N, and O from XPS results for CQDs, CN, and CQD/CN.

| **Samples** | **C (at%)** | **N (at%)** | **O (at%)** |
| --- | --- | --- | --- |
| **CQDs** | 73.68 | 21.66 | 4.66 |
| **CN** | 42.98 | 54.26 | 2.75 |
| **CQD/CN** | 42.30 | 55.38 | 2.32 |

**Table S2.** Dynamics analysis of emission decays of as-prepared samples.

| Sample | A_1_ | τ_1_ (ns) | A_2_ | τ_2_ (ns) | A_3_ | τ_3_ (ns) | τ_ave_ (ns) |
| --- | --- | --- | --- | --- | --- | --- | --- |
| CN | 3874.14 | 5.03448 | 11836.90 | 1.53152 | 183.2734 | 22.64514 | 5.26389 |
| CQD/CN | 3236.9297 | 6.13803 | 13480.416 | 1.58217 | 359.99713 | 27.59949 | 8.40795 |

The TRPL attenuation data were fitted using a triexponential function (Equ. 4), and the average lifetime of charge carriers was calculated (Equ. 5).

I(t) = A_1_exp (-t/τ_1_) + A_2_exp (-t/τ_2_) + A_3_exp (-t/τ_3_) (8)

τ_ave_ = (A_1_τ_1_^2^ + A_2_τ_2_^2^+ A_3_τ_3_^2^)/(A_1_τ_1_ + A_2_τ_2_ + A_3_τ_3_) (9)

Where A stands for relative amplitude and τ is the fluorescence lifetime.

**Table S3.** Comparison of photocatalytic CO and CH_4_, reduction activities for photocatalysts in this work and those reported in the literature.

| Photocatalyst | Reducing agent | Light source | CO yield (µmol·g^-1^·h^-1^) | CH_4_ yield (µmol·g^-1^·h^-1^) | Ref. |
| --- | --- | --- | --- | --- | --- |
| Alkyl group-decorated g-C_3_N_4_ | water vapor | 300 W Xe | 19.77 | 1.09 | ^[9]^ |
| TiO_2_/C_3_N_4_/Ti_3_C_2_ | water vapor | 350 W Xe | 4.39 | 1.20 | ^[10]^ |
| Cu/CN-0.25 | water vapor | 300 W Xe | 11.21 | 0.61 | ^[11]^ |
| Ti_3_C_2_/g-C_3_N_4_ | water vapor | 300 W Xe | 5.19 | 0.044 | ^[12]^ |
| B-doped g-C_3_N_4_ | water vapor | 300 W Xe | 0.45 | 0.16 | ^[13]^ |
| g-C_3_N_4_/Bi/CDs | water vapor | 300 W Xe | 9.08 | 0.188 | ^[14]^ |
| Cu_2_ZnSnS_4_/Pt-integrated g-C_3_N_4_ | water vapor | 400 W Xe | 17.351 | 7.961 | ^[15]^ |
| a-Mo/C_3_N_4_ | water vapor | 300 W Xe | 18 | 37 | ^[16]^ |
| NiFe-LDH/CN | water vapor | 300 W Xe | 55.1 | --- | ^[17]^ |
| IL/Co-bCN | water vapor  (3 mL H_2_O) | 300 W Xe | 40.5 | 6.3 | ^[18]^ |
| NiAl-LDH/CN/GA-20 | water vapor  (5 mL H_2_O) | 300 W Xe | 28.83 | ~1.3 | ^[19]^ |
| porous tubular yolk-shell g-C_3_N_4_ | water vapor  (1mL H_2_O) | 300 W Xe | 40.08 | --- | ^[20]^ |
| CoZnAl-LDH/RGO/g-C_3_N_4_ | water vapor  (0.4 mL H_2_O) | 300 W Xe | 10.11 | --- | ^[21]^ |
| p-C_3_N_4_/InVO_4_ | water vapor  (5 mL H_2_O) | 300 W Xe | 14.05 | --- | ^[22]^ |
| OH-g-C_3_N_4_/Bi_2_O_2_CO_3_ | water vapor | 450 nm LED  (4×3 W) | 26.69 | --- | ^[23]^ |
| Pt/g-C_3_N_4_ | water vapor  (0.1 mL H_2_O) | LED | 14.8 | --- | ^[24]^ |
| g-C_3_N_4_/LDH | 10 mL water | 300 W Xe | 29.29 | 1.32 | ^[25]^ |
| SnS_2_/g-C_3_N4 | 100 mL water | 300 W Xe | 40.86 | --- | ^[26]^ |
| CQD/CN | **water vapor** | **300 W Xe** | **47.84** | --- | **This work** |

**Supplementary Reference**

[1] W. Hou, H. Guo, M. Wu, L. Wang, *ACS Nano* **2023**, 17, 20560−20569.

[2] G. Kresse, J. Furthmüller, *Phys. Rev. B* **1996**, 54, 11169.

[3] P. E. Blöchl, *Phys. Rev. B* **1994**, 50, 17953.

[4] G. Kresse, D. Joubert, *Phys. Rev. B* **1999**, 59, 1758.

[5] J. P. Perdew, K. Burke, M. Ernzerhof, *Phys. Rev. Lett.* **1996**, 77, 3865.

[6] S. Grimme, J. Antony, S. Ehrlich, H. Krieg, *J. Chem. Phys.* **2010**, 132, 154104.

[7] J. K. Nørskov, J. Rossmeisl, A. Logadottir, L. Lindqvist, J. R. Kitchin, T. Bligaard, H. Jónsson, *J. Phys. Chem. B* **2004**, 108, 17886.

[8] V. Wang, N. Xu, J. Liu, G. Tang, W. Geng, *Comput. Phys. Commun.* **2021**, 267, 108033.

[9] C. Yang, Y. Hou, G. Luo, J. Yu, S. Cao, *Nanoscale* **2022**, 14, 11972.

[10] F. He, B. Zhu, B. Cheng, J. Yu, W. Ho, W. Macyk, *Appl. Catal. B* **2020**, 272, 119006.

[11] J. Wang, T. Heil, B. Zhu, C. W. Tung, J. Yu, H. M. Chen, M. Antonietti, S. Cao, *ACS Nano* **2020**, 14, 8584.

[12] C. Yang, Q. Tan, Q. Li, J. Zhou, J. Fan, B. Li, J. Sun, K. Lv, *Appl. Catal. B* **2020**, 268, 118738.

[13] J. Fu, K. Liu, K. Jiang, H. Li, P. An, W. Li, N. Zhang, H. Li, X. Xu, H. Zhou, D. Tang, X. Wang, X. Qiu, M. Liu, *Adv. Sci.* **2019**, 6, 1900796.

[14] X. Zhao, J. Li, X. Kong, C. Li, B. Lin, F. Dong, G. Yang, G. Shao, C. Xue, *Small* **2022**, 18, e2204154.

[15] A. Raza, H. Shen, A. A. Haidry, *Appl. Catal. B* **2020**, 277, 119239.

[16] R. Zhang, P. Li, F. Wang, L. Ye, A. Gaur, Z. Huang, Z. Zhao, Y. Bai, Y. Zhou, *Appl. Catal. B* **2019**, 250, 273.

[17] B. Zhu, Q. Xu, X. Bao, H. Yin, Y. Qin, X.-C. Shen, *Chem. Eng. J.* **2022**, 429, 132284.

[18] Y. Liu, J. Sun, H. Huang, L. Bai, X. Zhao, B. Qu, L. Xiong, F. Bai, J. Tang, L. Jing, *Nat. Commun.* **2023**, 14, 1457.

[19] M. Yang, P. Wang, Y. Li, S. Tang, X. Lin, H. Zhang, Z. Zhu, F. Chen, *Appl. Catal. B* **2022**, 306, 121065.

[20] N. Tian, K. Xiao, Y. Zhang, X. Lu, L. Ye, P. Gao, T. Ma, H. Huang, *Appl. Catal. B* **2019**, 253, 196.

[21] Y. Yang, J. Wu, T. Xiao, Z. Tang, J. Shen, H. Li, Y. Zhou, Z. Zou, *Appl. Catal. B* **2019**, 255, 117771.

[22] L. Wang, D. Chen, S. Miao, F. Chen, C. Guo, P. Ye, J. Ning, Y. Zhong, Y. Hu, *Chem. Eng. J.* **2022**, 434, 133867.

[23] J. Li, C. He, N. Xu, K. Wu, Z. Huang, X. Zhao, J. Nan, X. Xiao, *Chem. Eng. J.* **2023**, 452, 139191.

[24] N. Sun, Y. Zhu, M. Li, J. Zhang, J. Qin, Y. Li, C. Wang, *Appl. Catal. B* **2021**, 298, 120565.

[25] R. Wang, Z. Wang, Z. Qiu, S. Wan, J. Ding, Q. Zhong, *Chem. Eng. J.* **2022**, 448, 137338.

[26] Y. Li, Q. Yin, Y. Zeng, Z. Liu, *Chem. Eng. J.* **2022**, 438, 135652.
